# An effective AI model for automatically detecting arteriovenous fistula stenosis

**DOI:** 10.1038/s41598-023-35444-6

**Published:** 2023-10-17

**Authors:** Wheyming Tina Song, Chang Chiang Chen, Zi-Wei Yu, Hao-Chuan Huang

**Affiliations:** 1 Deparment of Information Engineering and Computer Science, Feng Chia University, Taichung, Taiwan; 2 Department of Industrial Engineering and Engineering Management, National Tsing Hua University, Chinchu, Taiwan; 3 National Taiwan University Hospital Hsin-Chu Branch, Hsinchu, Taiwan

**Keywords:** Computational biology and bioinformatics, Health care, Medical research

## Abstract

In this study, a novel artificial intelligence (AI) model is proposed to detect stenosis in arteriovenous fistulas (AVFs) using inexpensive and non-invasive audio recordings. The proposed model is a combination of two new input features based on short-time Fourier transform (STFT) and sample entropy, as well as two associated classification models (ResNet50 and ANN). The model’s hyper-parameters were optimized through the use of the design of the experiment (DOE). The proposed AI model demonstrates high performance with all essential metrics, including sensitivity, specificity, accuracy, precision, and F1-score, exceeding 0.90 at detecting stenosis greater than 50%. These promising results suggest that our approach can lead to new insights and knowledge in this field. Moreover, the robust performance of our model, combined with the affordability of the audio recording device, makes it a valuable tool for detecting AVF stenosis in home-care settings.

## Introduction

ACCORDING to 2021 USRDS^1^, over one million dialysis patients worldwide undergo hemodialysis to treat end-stage renal disease. To perform dialysis, vascular access is essential for allowing easy cannulation and providing a high blood flow. The arteriovenous fistula (AVF), a surgical connection between an artery and a vein, is the preferred choice among all possible vascular accesses. However, AVF can sometimes become dysfunctional due to conditions such as stenosis, which is a narrowing of the pathway. In severe cases, AVF stenosis can lead to potentially life-threatening complications.

Effective AVF-stenosis detection treatments, typically used in hospitals, include percutaneous transluminal angiography (PTA) and color duplex imaging (CDI, also known as ultrasound). These treatments require expensive equipment and professional medical personnel, making them unsuitable for routine screening. To address these limitations, non-invasive devices have been developed to record vascular sounds, and various studies have investigated automatic AVF-stenosis detection.

**Figure 1:**
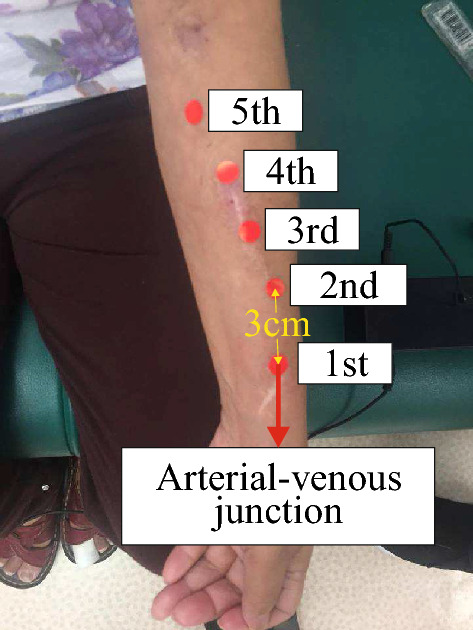
Five positions.

This paper aims to present a reliable AI model for AVF-stenosis detection using time-domain vascular sounds collected in a home-care setting. The study recorded vascular sounds from forty patients with mature AVFs at five positions along the vein (see Fig. 1). The first position was the arterial-venous junction, and the subsequent positions were spaced approximately 3 cm apart, as recommended by the second author who is an experienced nephrologist. The total recording time for all 5 positions was approximately 30 minutes. The selection of these 5 positions was based on the observation that stenosis in AVFs tends to occur most frequently at the juxta-anastomotic site or at the outflow vein, as reported in^2^. The purpose of selecting only five positions for the vascular-sounds collections was to provide a representative sample while minimizing the waiting time for patients.

**Figure 2:**
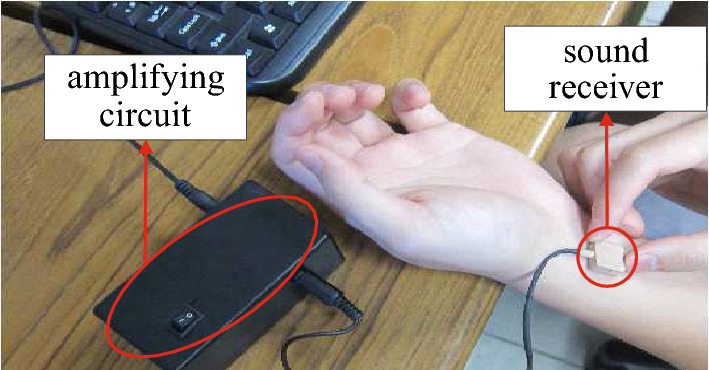
The recording device.

The audio recording equipment used was an electronic stethoscope (see Fig. 2). The recording device consists of an amplifying circuit and a sound receiver (also named a condenser microphone), which is protected by insulating material. The audio recording device was developed by the Taiwan Industrial Technology Research Institute (ITRI), and had an affordable price, costing less than USD 30.

**Figure 3:**
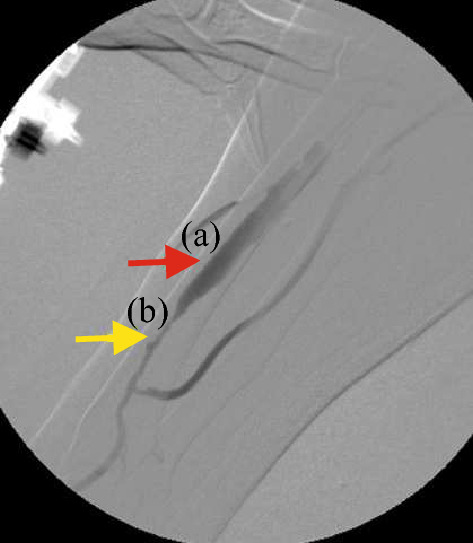
An angiography photo: (**a**) 0% reduction, (**b**) 80% reduction.

In this research study, data was collected from dialysis patients before and after they underwent a PTA procedure. The PTA records showed that at least one blood vessel of the five positions examined with a significant reduction in diameter, as determined by nephrologists using angiography and comparison with an adjacent vessel or graft. This significant reduction, known as vascular stenosis, was defined as being greater than 50%. In an angiography image, shown in Fig. 3, point (a) shows a 0% reduction (marked with a red arrow), while point (b) shows an 80% reduction (marked with a yellow arrow). After a PTA procedure, none of the patients had significant stenosis.

There was a total of 153 original time-domain data, with 73 abnormal and 80 normal. For each position of each patient, we collected the vascular sounds at a rate of 22050 per second for a duration 60 seconds.

The remainder of this paper is organized as follows. The review of the relevant literature, consisting of 14 archived publications, is presented in “literature review” section. The proposed methods for data pre-processing, input features, and classification AI models are described in proposed data pre-processing, proposed input features, and proposed AI model sections, respectively. Section “performance and discussion” presents the performance results of the proposed AI model, as well as a comparison of its performance with that of 14 previously published studies. The conclusion and suggestions for future research are presented in “conclusion and future research” section.

## Literature review

Numerous research studies have endeavored to create an automated mechanism that can detect stenosis in AVFs through the use of non-invasive tools that capture vascular sound. The earliest works in this field date back to references^3–6^. More recent studies since 2014, which include fourteen published papers on automated detection of AVF stenosis, are outlined in Table 1.

**Table 1:** Literature Review (Two proposed AI Models are marked in bold at bottom).

C1	C2	C3	C4	C5	C6	C7	C8	C9	C10	C11	C12	C13	C14	C15
No	Ref.	Year	Num Patient	Cardiac Cycles Total (S,H)	Filter- ing	Input feature. 1.Image. 2.Texture. 3. Both	Data Transformation	Classifier	DOE	Sen. (SE)	Spe. (SE)	Acc. (SE)	Pre. (SE)	F1-S. (SE)
1	^7^	2014	74	878 (443 ,435)	TF	2	S Trans.	RBF-NN	–	0.89	−	−	0.87	0.88
2	^8^	2016	19	1190 (− ,−)	TF	2		KNN	–	–	−	−	−	0.97
3	^2^	2017	−	−	TF	2	MEM PCA	SOM	–	–	−	0.7	−	−
4	^9^	2017	21	42*n* *n* not clear	TF	2	STFT	Fuzzy Petri Nets	−	−	−	0.86	−	−
5	^10^	2018	20	−	TF	2	CWT	−	−	−	−	−	−	−
6	^11^	2018	60	300*n* *n* not clear	−	2	FT	SVM	−	−	−	0.55	− −	0.55 −
7	^12^	2018	24	3283 (− ,−)	−	2	CWT LPC	−	−	−	−	−	− −	− −
8	^13^	2018	−	−	EMD	2	Hilbert	−	−	−	−	−	−	−
9	^14^	2019	−	−	−	2	CWT LPC	−	−	−	−	−	− −	− −
10	^15^	2019	−	3441 (− ,−)	−	2	CWT	Threshold based Classifier	−	0.91	0.92	0.90	− − −	− − −
11	^16^	2019	38	T1: −	−	2	WT	KNN	−	−	−	−	0.93	0.91
				T2: −					−	−	−	0.81	0.71	0.71
				T3: −					−	−	−	−	0.66	0.69
				T4: −					−	−	−	−	0.81	0.78
				T5: −					−	−	−	−	0.81	0.81
				T6: −					−	−	−	−	0.92	0.92
12	^17^	2020	20	$V_1$: 879	−	1	FT	CNN	−	0.49	0.84	0.75	0.45	0.46
				$V_2$:1479					−	0.86	0.81	0.83	0.77	0.81
				$V_3$:1233					−	0.48	0.82	0.73	0.48	0.48
				$V_4$: 95					−	0.51	0.94	0.93	0.01	0.03
				$V_5$: 308					−	0.58	0.89	0.86	0.36	0.44
13	^18^	2021	46	(−, −)	−	2	FFT	SVM	−	0.91	0.99	0.98	−	−
14	^19^	2022	40	(−,0)	−	1	STFT	RN50		0.95	−	−	0.95	0.96
15	This Paper	2023	40	1510 (730, 780)	EMD	3	$\bullet$ STFT (image)	$\bullet$ RN50	$\checkmark$	0.91 (0.01)	0.91 (0.01)	0.90 (0.01)	0.91 (0.01)	0.91 (0.01)
							$\bullet$**Sample**	$\bullet$ ANN						
							**-Entropy**	$\bullet$ **Concat.**						

To ensure comprehensiveness, the present article is included in Table 1, appearing at the bottom of the table with the new suggestion highlighted in bold. In instances where the corresponding information is not explicitly presented in the cited paper, Table 1 represents this with a hyphen “−”’.

Table 1 contains several columns, where C1 to C5 represent the paper number, referenced index, publication date, number of patients, and the total number of AVF-stenosis (S) and normal (N) cardiac cycles. The degree of AVF-stenosis is categorized differently in various studies.^16^ categorized it into six types (T1-T6), while^17^ classified the collected vascular sounds as 5 types: normal, hard, high, intermittent, whistle; denoted as V1−V5 respectively.

C6 and C7 of Table 1 represent the adopted filtering method and input features used in the studies, respectively. Traditional filtering (TF) and Empirical Mode Decomposition (EMD) were the two filtering methods used. The input features were categorized into three types: image (1), texture (2), and both (3). Our study stands out from the fourteen publications listed in Table 1 as we used a unique approach by combining two types of input features, potentially leading to new insights and knowledge in this field.

C8 of Table 1 displays the data transformation methods used in the studies. The listed abbreviations include S-transform, MEM (maximum entropy method)^20^, PCA (principal component analysis)^21^, FT (fourier transform)^22^, FFT (fast fourier transform)^22^, STFT (short-time fourier transform)^22^, WT (wavelet transform)^22^, CWT (Continuous wavelet transform), and LPC (Linear predictive coding). It is important to note that the STFT used in this work and reference^9^ was presented as an image, whereas in reference^9^, it was presented in the form of a texture.

C9 of Table 1 displays the data classifiers used in the studies. The listed classifiers include RBF-NN (radical basis function neural network)^23^, KNN (k-nearest neighbors)^24^, SOM (self-organizing maps)^25^, LSTM (long short-term memory)^26^, FPN (fuzzy petri nets)^27^, SVM (support vector machines)^28^, and CNN (convolutional neural networks)^29^.

C10 of Table 1 displays the use of the DOE method. It is important to note that none of the listed fourteen publications mentioned using the DOE method to optimize the hyper-parameters of the adopted models. C11 to C15 represent the five performance measures adopted in this study, along with their associated standard errors (SE): sensitivity, specificity, accuracy, precision, and F1-Scores (F1-S), respectively. It’s worth noting that SE is utilized as a quality measure for assessing the accuracy of these performance estimates. A different technique for conveying the significance of digits in these estimated performance values involves the utilization of the leading digit rule (LDR), as explained in the references cited under^30,31^. The information provided in Table 1 demonstrates that, with the exception of this article, none of the other 14 references include SE in their reporting of these performance measures.

Although study^19^ reported a high sensitivity of over 0.95 for venous outflow stenosis detection, the lack of specificity and potential selection bias due to a single interventional radiologist performing interventions on all studied data may weaken the reliability of the results.

The performance of the published works is influenced by the analysis methods used, as well as the type of non-invasive audio recordings used. Study^18^, for instance, utilized a pulse radar sensor, which is different from our approach using an inexpensive audio recording device. However, the purpose of this paper is to evaluate the effectiveness of the adopted analysis methods, rather than examining the impact of different non-invasive audio recordings.

## Proposed methodology

**Figure 4:**
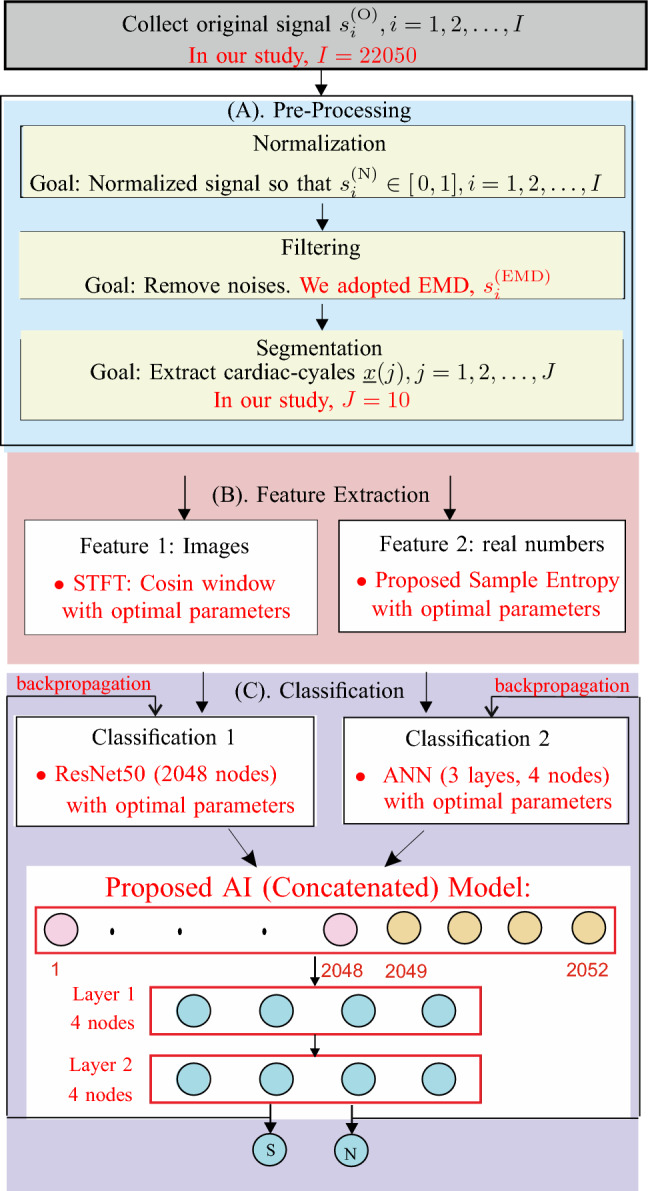
Proposed AI (Concatenated) model.

The process of automatically classifying AVF-stenosis typically involves three steps: (A) pre-processing, (B) features extraction, and (C) classification using deep learning models. Pre-processing, involves preparing the data for analysis. In Step (B), relevant features were identified and extracted from cardiac-cycles, obtained in Step (A). These features can be divided into two categories: (1) visual features represented as images, and (2) textual features represented as real numbers. Table 1 shows that most of the published research on this topic has focused on textual features, with only one study,^19^, utilizing visual features. In Step (C), associated classification models based on the two types of features were selected. The final binary results were designated as stenosis (S) or normal (N). Figure 4 illustrates the general three-step process in black, with the specific methods adopted in our study highlighted in red. We used Python version 3.6.10 in our analysis.

### Proposed data pre-processing

The proposed data pre-processing method consists of four tasks: normalization, EMD filtering, segmentation, and data augmentation. In detail:

- *Normalization*: This task involves adjusting the range and distribution on the data to be within [0, 1]. The normalized data, denoted as $s_i^{(\mathrm N)}$, is written as a function of the original data, denoted as $s_i^{(\mathrm O)}$.
  1 $$\begin{aligned} s_i^{(\mathrm N)} = \dfrac{s_i^{(\mathrm O)}- a}{b- a}, i = 1, 2, \ldots , I, \end{aligned}$$
  where $$a = {\textrm{min}} (s^{(\textrm{O})}_{i}, i = 1, 2, \ldots , I )$$, $$b = {\textrm{max}} (s^{(\textrm{O})}_{i}, i = 1, 2, \ldots , I )$$, and $$I = 22050 \times 60$$.
- *EMD filtering*: This task involves using the Empirical Mode Decomposition (EMD)^32^ technique to extract useful information from the data. The proposed EMD filtering process, denoted as $s^{(\textrm{EMD})}$, includes two major steps: (1) decompose $s_i^{(\mathrm N)}$ into a finite number of intrinsic mode functions (IMFs, denoted as $c_i(j), j=1, 2, 3, \ldots$) and a residual. (2) remove unimportant IMFs. The study in our work shows that removing the first IMF provides the optimal performance; $s^{(\textrm{EMD})}_i = s_i^{(\mathrm N)}-c_i(1)$.
- Segmentation: This step involves dividing the data into smaller section or segments for easier analysis. In this study, we extracted 10 cardiac-cycles from each signal $s^{(\textrm{EMD})}_i, i=1, 2, \ldots 22050$.

### Proposed input features

Two input features are discussed: the short-time fourier transform (STFT) and a modified version of sample entropy with optimized parameters (referred to as “proposed sample entropy”).

#### Short-time fourier transform (STFT)

Before delving into the topic of STFT, let’s briefly visit its foundation, the Fourier transform (FT), which transforms signals from the time domain $x_{i}(j)$ to the frequency-domain FT(*k*).

2 $$\begin{aligned} {\textrm{FT}}(k) &= \sum _{i=1}^{L_j}x_{i}(j) \\ & \quad \times \exp (-\sqrt{-1}(k-1)\frac{2\pi }{L_j}(i-1)). \end{aligned}$$

As a university teacher with 30 years of experience, I have noticed that despite students being able to write the equation of the FT, they often struggle to understand its true purpose and insight in signal analysis. This is due to the complexity of the mathematical equations associated with the FT.

**Figure 5:**
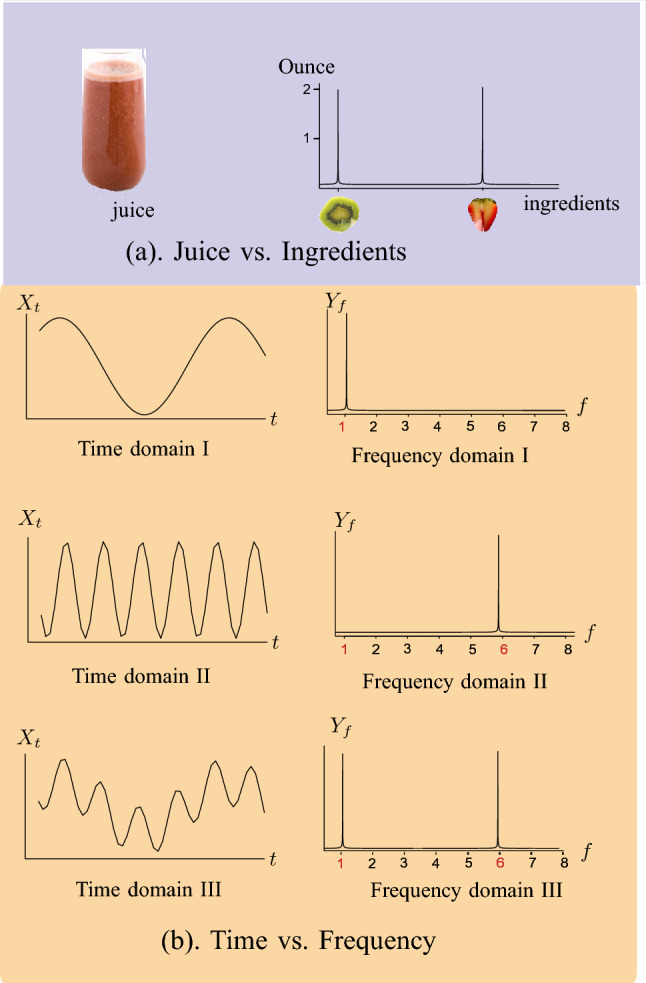
(**a**) Juice vs. Ingredients; (**b**) Time vs. Frequency.

The metaphor of “juice vs. recipe”, illustrated in Fig. 5, can be useful in explaining the purpose and insight of the FT. Figure 5a represents juice vs. ingredients (kiwi and strawberry, 2 ounces of each) and Fig. 5b represents time-domain signal vs. frequency-domain signal. Just as it is simpler to understand the components of a juice by analyzing each ingredient separately rather than the blended juice as a whole, the same principle applies to analyzing signals. Examining each frequency component individually is easier than trying to comprehend the signals in its time domain form.

Now we will be focusing on STFT. The STFT is a sequence involving the FT of a windowed signal. where FT converts data from a time-domain to a frequency domain. Specifically, STFT first divides a longer time signal into shorter segments of equal length (window width) and then computes the FT separately on each shorter segment. The definition of the STFT with a window function *w*, and a window width *m* is shown in Eq. (3),

3 $$\begin{aligned} \begin{aligned}{}&{\textrm{STFT}}(x_i(j), k, m) = \sum _{i=1}^{L_j}x_{i}(j) w(i-m) \\ \times&\exp (-\sqrt{-1}(k-1)\frac{2\pi }{L_j}(i-1)). \end{aligned} \end{aligned}$$

**Figure 6:**
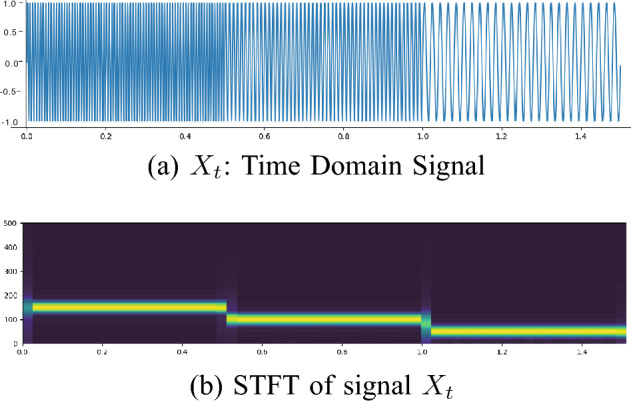
Time signal vs. the associated STFT.

The advantage of STFT allows us to perform time and frequency analysis in the same plot, in which the *x*-axis represents the time and y-axis represents the frequency .

To provide readers a quick view of STFT, we define a signal $X_t, t \in [0,1.5]$, as shown in Eq. (4). The plots of $X_t$ and its corresponding STFTs are depicted in Fig. 6(a) and (b).

4 $$\begin{aligned} X_t = \left\{ \begin{array} {lll} \textrm{cos}(2 \pi 150 t), &{} 0< t \le 0.5 ~ \\ \textrm{cos}(2 \pi 100 t), &{} 0.5< t \le 1~ \\ \textrm{cos}(2 \pi 50 t), &{} 1 < t \le 1.5~ \end{array} \right. \end{aligned}$$

#### Proposed sample entropy with optimal parameters

Sample Entropy (denoted as $\textrm{S}_{\textrm{E}}$), which has not been investigated in detecting AVF-stenosis, is defined in Eq. (5).

5 $$\begin{aligned} \textrm{S}_{\textrm{E}}({\underline{z}},m,r) = - \textrm{log}(A/B), \end{aligned}$$

which is the negative natural logarithm of the conditional probability that A given B. The parameters A, B, three arguments (${\underline{z}},m,r$), and related notations used in Eq. (5) are defined below.

- ${\underline{z}}$ contains the time domain data ${\underline{x}}$ that satisfies the transformed frequencies $f \in (F_{\textrm{L}}, F_{\textrm{H}})$, where $F_{\textrm{L}}$ and $F_{\textrm{H}}$ are pre-determined lower and upper parameters for the frequency domain signal, respectively.
- $${\underline{z}}^{(m)}(i) = \{ z_{i}, z_{i+1}, \ldots , z_{i+m-1}\}$$
- $d[{\underline{z}}^{(m)}(i), {\underline{z}}^{(m)}(j)]$. $\equiv \max \{ |g-h|, g \in {\underline{z}}^{(m)}(i), h \in {\underline{z}}^{(m)}(j) \}$, where *g*, *h* are coordinated elements in ${\underline{z}}^{(m)}(i)$ and ${\underline{z}}^{(m)}(j)$, respectively. That is, $d[{\underline{z}}^{(m)}(i), {\underline{z}}^{(m)}(j)]$ is the greatest differences along any coordinate dimension.
- $\textrm{sd}({\underline{z}})$ is the standard deviation of all elements in ${\underline{z}}$.
- The notation *A* is the number of template vector pairs having $d[{\underline{z}}^{(m)}(i), {\underline{z}}^{(m)}(j)] < r \cdot \textrm{sd}({\underline{z}}), i \ne j$; where *r* is a parameter.
- The notation *B* is the number of template vector pairs having $d[{\underline{z}}^{(m+1)}(i), {\underline{x}}^{(m+1)}(j)] < r \cdot \textrm{sd}({\underline{z}}), i \ne j$. where ${\underline{z}}^{(m+1)}(i) = \{ z_{i}, z_{i+1}, \ldots , z_{i+m}\}$

The proposed sample entropy $\textrm{S}_{\textrm{E}}$, defined in Eq. (5), is an extension of the traditional sample entropy, which can be used to measure the complexity of time-series signals such as vascular sounds. Sample entropy with large values indicates irregular signals whereas those with smaller values characterize more regular signals. For example. Consider two series (a) 1, 2, 1, 2, 1, 2, 1, 2, 1,2; and (b) 1, 1, 2, 1, 2, 2, 2,1, 1, 2. Both series have the same mean and variance, but different sample entropies. Specifically, the sample entropy ($m=2$) for series (a) and (b) are 0 and 1.386294, respectively. It is simple to deduce that series (a) has a more consistent pattern compared to (b).

In the proceeding discussion, we will explore the process through which we determined the optimal parameters for the $\textrm{S}_{\textrm{E}}$, defined in Eq. (5). A comprehensive design of experiment (DOE) was carried out utilizing four factors, each possessing varying levels.

- Factor 1: 3 levels. $$F_{\textrm{L}} = 10, 50, 100.$$
- Factor 2: 9 levels. $$F_{\textrm{H}} = 200, 300, \ldots , 1000.$$
- Factor 3: 9 levels. $$m = 2, 3, \ldots , 10.$$
- Factor 4: 5 levels. $$r = 0.1, 0.2, \ldots , 0.5.$$

From the 1215 ($3 \times 9 \times 9 \times 5 =1215$) possible combinations of levels, the best five combinations illustrated in Table 2, showed accuracy, sensitivity, and specificity all above 0.70. The best combination utilized the parameters $F_{\textrm{L}} =10$, $F_{\textrm{H}} =700$, $m =4$, and $r=0.2$.

**Table 2:** Top five combinations for $S_{\textrm{E}}$.

Rank	*F*_L_	*F*_H_	*m*	*r*	Sensitivity	Specificity	Accuracy
1	10	700	4	0.2	0.73	0.73	0.72
2	10	500	7	0.3	0.72	0.72	0.71
3	10	1000	5	0.2	0.71	0.72	0.72
4	10	1000	4	0.3	0.71	0.70	0.70
5	10	600	4	0.3	0.70	0.70	0.70

**Figure 7:**
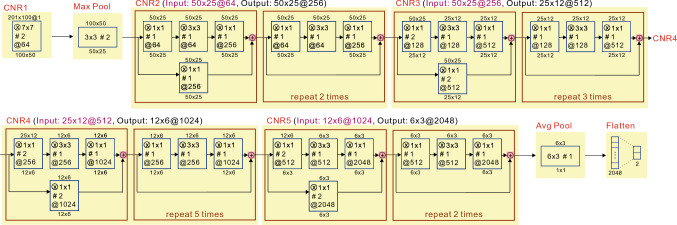
visual representation of the ResNet50 framework.

### Proposed AI model

This section proposes a concatenated AI model (combining ResNet50 and ANN) that incorporates optimal hyper-parameters determined through DOE. ResNet50 is the dominant component of the proposed model and will be further discussed in “proposed AI model” section. The optimal hyper-parameters will be discussed in “proposed AI model” section.

#### Insight of ResNet50

ResNet50^33^ is a type of deep learning model called a residual neural network,which consists of 50 convolutional layers. The innovation of ResNet lies in addressing the vanishing gradients problem. This problem can make the computation of the loss function difficult. This is achieved through the use of residual connections, which allow the gradients to flow more easily through the model during training.

The proposed graph in Fig. 7 provides an informative and enlightening graph showing the structure of ResNet50. The Notation used in the graph, including $\otimes$ , #, and $\oplus$, is explained below.

- Notation $\otimes$: indicates the dimension of the kernel function. Ex. “$\otimes$3x3” indicates that the kernel dimension is 3x3, which has 9 parameters.
- Notation #: indicates the stride used in convolution function. Ex. “#2” indicates that each convolution is done with moving two steps.
- Notation @: indicates the number of images. Ex. “@25” indicates that there are 25 images.
- Notation$\oplus$: indicates the addition of two associated pixel in the same location. Ex.: “5x5@25$\oplus$5x5@25 $=$5x5@25” indicates that the total number of images (25) remain the same, but the pixel of each associated image after $\oplus$ changes.

In Fig. 7, the main components highlighted in red are labeled as CNR1, Maxpool, CNR2, $\ldots$, and CNR5, where CNR stands for convolution, normalization, and ReLu.

The number of convolutional layers in ResNet50 can be determined using Eq. (6).

6 $$\begin{aligned} \begin{aligned} 50 =&1+1+[3+(3\times 2)]+[3+(3\times 3)] \\&+[3+(3\times 5)]+[3+(3\times 2)], \end{aligned} \end{aligned}$$

where the 1st 1 listing in the right-hand side of Eq. (6) denotes for the one convolutional layer used in CNR1, the 2nd 1 denotes for the one convolutional layer used in MaxPool. The following $[3+(3\times 2)] = 9$, $[3+(3\times 3)] = 12, [3+(3\times 5)] =18$, and $[3+(3\times 2)]=9$ denote for the associated convolutional layer used in CNR2 to CNR5, respectively.

#### Proposed AI model with optimal hyper-parameters

The input-feature part of the proposed AI Model incorporates STFT and the proposed sample entropy. The classification part of the model is formed by combining ResNet50 and ANN into one structure. The optimal hyper-parameters for the proposed AI model will be derived in terms of accuracy through the use of $2^{5-1}$ fractional-factorial design on six chosen factors, named A to E.

- Factor $\textrm{A}$ (No. of layers in ANN) with 2 levels: $-1$ (2 layers), 1 (3 layers)
- Factor $\textrm{B}$ (No. of nodes in ANN) with 2 levels: $-1$ (4 nodes), 1 (10 nodes)
- Factor $\textrm{C}$ (TF vs. EMD) with 2 levels: $-1$ (TF), 1 (EMD), where TF dentes for the traditional filtering method and EMD
- Factor $\textrm{D}$ (No. of layer in concatenated model): $-1$ (2 layers), 1 (4 layers)
- Factor $\textrm{E}$ (No of nodes in concatenated model): $-1$ (4 nodes), 1 (10 nodes)

In Table 3, the p-values associated with main factors (A to E) and interaction BD are displayed in the the rightmost column of Table 3. The p-values for all the factors are extremely small, specifically 0.000. This implies that the associated factors are significant and play a crucial role in optimizing the performance of our proposed model.

**Table 3:** Concatenated, main effect: accuracy.

Factor	Effect	Coef	T-Value	*P*-value
A	− 0.055	− 0.275	− 64.41	0.000
B	− 0.036	− 0.018	− 42.45	0.000
C	0.180	0.089	207.87	0.000
D	− 0.020	− 0.011	− 24.89	0.000
E	0.040	0.028	46.84	0.000
B * D	0.030	0.015	35.13	0.000

### Ethical approval

All experimental protocols were approved by IRB/ethics committee of Taiwan University Hospital Hsin-Chu Branch.

All procedures performed in studies involving human participants were in accordance with the ethical standards of the institutional and/or national research committee and with the 1964 Helsinki declaration and its later amendments or comparable ethical standards.

- Protocol Title: Detection of arteriovenous shunt stenosis by a portable vascular sounds measuring device
- Research/ Trial Institution: National Taiwan University Hospital Hsin-Chu Branch
- Department/ Principal Investigator: Department of Internal Medicine/ Dr. Chang-Chiang Chen
- Informed consent form Version/ Date: Version 3, 2020-7-13

### Regulations for methods

All methods were carried out in accordance with relevant guidelines and regulations.

### Informed consent

Informed consent was obtained from all individual participants included in the study.

## Performance and discussion

The proposed concatenated AI model utilizes ResNet 50 and ANN as classification models and combines image and texture features. Table 4 presents the top five combinations of this model along with their corresponding results. The standard errors (SE) for the estimated performances are provided within parentheses, and all of them are below 0.02. The combination highlighted in red achieved impressive results with all evaluation metrics exceeding 0.9. The best combination for the six factors were: (A) hidden layers 2 for ANN, (B) layer nodes 4 for ANN, (C) EMD as the filtering method, (D) hidden layers 2 for the concatenated model, and (E) layer nodes 4 for the concatenated model. The fact that the top five combinations of this model all include using EMD suggests that EMD is a crucial technique for achieving optimal performance in the proposed concatenated model.

After evaluating our proposed AI model against 14 publications listed in Table 1 using accuracy, sensitivity, precision, and F1-score metrics, we discovered that it was outperformed by studies^18^ and^19^. It is worth noting, however, that study^19^ may lack specificity and could be subject to selection bias, which reduces the reliability of their results. Study^18^, on the other hand, used a pulse radar sensor that differs from our approach that uses an inexpensive audio recording device. Moreover, it is unclear what the stenosis-to-normal data ratio was in their study. Therefore, it is not entirely clear whether studies^18^ and^19^ outperformed our proposed AI model in this study.

In summary, our proposed AI model demonstrated promising results, with all five metrics exceeding 0.90 for detecting stenosis greater than 50%. By utilizing a unique approach and comparing our results with those of previous studies, our research provides valuable insights into this field’s literature.

**Table 4:** Top Five Combinations for the Concatenated Model.

Rank	A	B	C	D	E	Sen. (SE)	Spe. (SE)	Acc. (SE)	Pre. (SE)	F1-S (SE)
1	2	4	EMD	2	4	0.91 (0.01)	0.91 (0.01)	0.90 (0.01)	0.91 (0.01)	0.91 (0.01)
2	2	4	EMD	4	4	0.91 (0.01)	0.90 (0.01)	0.90 (0.01)	0.90 (0.01)	0.90 (0.01)
3	3	4	EMD	2	4	0.90 (0.01)	0.90 (0.02)	0.89 (0.01)	0.89 (0.02)	0.89 (0.01)
4	3	100	EMD	4	100	0.87 (0.01)	0.86 (0.02)	0.86 (0.02)	0.86 (0.02)	0.85 (0.02)
5	2	100	EMD	2	100	0.86 (0.01)	0.86 (0.02)	0.85 (0.02)	0.84 (0.02)	0.86 (0.02)

## Conclusion and future research

The detection of AVF-stenosis is a critical research area in the diagnosis of end-stage renal disease. In this study, we propose a novel and effective AI model for detecting AVF-stenosis. Our proposed model concatenates “image feature” and “texture feature” using STFT (frequency-domain features) and sample entropy (time-domain features) with associated AI models (ResNet50 and ANN) and optimal hyper-parameters identified through DOE.

Our work makes a significant contribution by introducing a unique concatenation approach that has not been explored in the literature. Our experimental results demonstrate the effectiveness of our approach, with all evaluation metrics, including accuracy, sensitivity, specificity, precision, and F1-Score, surpassing the threshold of 0.90. These promising results suggest that our approach can lead to new insights and knowledge in this field.

As discussed in “literature review” section, the performance outcomes reported in the fourteen papers were influenced by the use of diverse non-invasive audio recording methods. To further enhance the applicability of our proposed concatenation approach, future research could explore its performance with a range of affordable non-invasive audio recording devices, considering that the results were dependent on the specific recording equipment used.

## Data Availability

The original data, time-domain signal before and after PTA (in txt and wav format), used in this research was upload to IEEE DataPort for your reference. IEEE DataPort. DOI: 10.21227/08qw-nk19. Citation Author: Wheyming Song. Data Format: .txt, .wav. Content: AVF-RESEARCH-TIME-DOMAIN-DATA-BEFORE-AFTER-PTA.
